# The effect of mindfulness-based stress reduction on presenteeism among ICU nurses: A *cluster randomized controlled trial*

**DOI:** 10.1371/journal.pone.0334825

**Published:** 2025-10-28

**Authors:** Xiaoli Liu, Shu Luo, Xianxiu Wen, Xiahong Huang, Jijun Wu, Min Chen, Ping Jia

**Affiliations:** 1 Department of critical Medicine, Deyang people’s Hospital, Sichuan, China; 2 Department of Psychosomatic Medicine, Deyang people’s Hospital, Sichuan, China; 3 Nursing Department of Sichuan Provincial people’s Hospital, Sichuan, China; 4 Neurological intensive Care Unit, Sichuan people’s Hospital, Sichuan, China; Universiti Pertahanan Nasional Malaysia, MALAYSIA

## Abstract

**Background:**

Due to high-pressure environments, heavy workloads, and working in “three-shift” schedules, Intensive Care Unit (ICU) nurses experience high-level presenteeism. This may compromise nursing quality and patient safety and damage nurses’ physical and mental health. Therefore, there’s an urgent need for effective interventions to promote the healthy development of nursing human resources and maintain nursing team stability.

**Aim:**

To evaluate the effect of an 8-week Mindfulness-Based Stress Reduction (MBSR) training on presenteeism among ICU nurses.

**Methods:**

ICU nurses with high levels of presenteeism were invited to participate in the study. The ICU wards were randomly assigned to either the intervention group or the control group. The intervention group (40 nurses) received an 8-week MBSR program delivered by a certified mindfulness therapist. In comparison, the control group (40 nurses) received standard psychological counseling, including emotional control, psychological regulation, and sleep management. Both groups were assessed using the Stanford Presenteeism Scale-6 (SPS-6) and the Five Facet Mindfulness Questionnaire (FFMQ) before and after the intervention, and 12 weeks after the intervention.

**Methods:**

This study employed a cluster randomized controlled trial with a two-arm design. ICU nurses with high presenteeism were invited and randomly assigned to groups by floor. The intervention group (40 nurses) underwent an 8-week MBSR program delivered by a certified mindfulness therapist, while the control group (40 nurses) received standard psychological counseling, including emotion regulation, psychological adjustment, and sleep management. Both groups were assessed using the Stanford Presenteeism Scale-6 (SPS-6) and the Five Facet Mindfulness Questionnaire (FFMQ) at baseline, post-intervention, and 12 weeks post-intervention.

**Results:**

*Linear mixed model* analysis showed significant group, time, and group-time interaction effects on SPS-6 scores (*P* < 0.05). The experimental group had significantly lower SPS-6 scores at 8 and 12 weeks post-intervention than the control group and their pre-intervention scores (*P* < 0.05). For FFMQ scores, significant group and time effects (*P* < 0.05) but no significant group-time interaction (*P* > 0.05) were found. The experimental group’s FFMQ scores were significantly higher at 8 and 12 weeks post – intervention than the control group and their pre-intervention scores (*P* < 0.05).

**Conclusion:**

The Mindfulness-Based Stress Reduction intervention was associated with increased mindfulness levels over time, and it significantly reduced presenteeism, with sustained effects observed over time.

**Clinical implications for nursing management:**

MBSR, as a psychological intervention method, has the advantages of improving nurses’ mental health and work efficiency, reducing presenteeism, and ensuring patient safety. Nursing managers can integrate MBSR into hospital policies by organizing regular MBSR sessions on mental health days or during team-building activities. This not only enhances nurses’ psychological resilience but also promotes a positive work environment, contributing to a safer and more efficient healthcare setting.

**Patient or Public Contribution:**

Participants were involved solely in the data collection process. No participant contributions were required for the study’s design, outcome measurement or implementation.

## 1 Introduction

Globally, the shortage of nursing human resources has become a major crisis in healthcare systems. According to data from the World Health Organization (WHO), the global nursing shortage is projected to reach 5.7 million by 2030. The Intensive Care Unit (ICU), which requires the highest level of specialized skills and immediate responsiveness, is facing particularly severe challenges [1]. ICU nurses bear core responsibilities such as life support for critically ill patients, operation of complex medical equipment, and high-risk decision-making, with their work quality directly impacting patient mortality and complication rates [2,3]. However, this group is chronically exposed to high-intensity work environment, emotional exhaustion, and traumatic events, leading to a high prevalence of physical and mental health issues and triggering presenteeism—a state in which nurses remain on duty but experience significant declines in work efficiency, cognitive ability, and emotional stability [2,4]. This “physically present but mentally absent” work state not only exacerbates the vicious cycle of global nursing shortages but also poses latent threats to patient safety [3,5].

Presenteeism is defined as “attending work while impaired by physical or psychological illness, resulting in reduced work effectiveness,” with core characteristics including attention deficits, increased decision-making errors, and prolonged task completion times [1,4]. In the nursing field, presenteeism rates are significantly higher than in other professions. Systematic reviews indicate a presenteeism rate of 35%−60% among general nurses. Meanwhile, ICU nurses, due to the unique demands of their work environment, exhibit rates as high as 53.9%−62% [1,6,7]. A survey of 722 ICU nurses in a public hospital in western China found that 53.9% exhibited high levels of presenteeism (SPS-6 score ≥16), which was significantly correlated with emotional exhaustion and low occupational self-efficacy [1].

The harms of presenteeism are twofold: For individual nurses, long-term work under physical or emotional strain exacerbates anxiety, depressive symptoms, chronic pain, and cardiovascular disease risks [1–3]; For healthcare systems, presenteeism is directly linked to increased medication error rates, failures in hospital infection control, and reduced patient satisfaction, with productivity losses reaching 3–7 times those of absenteeism [5,8,9]. For example, a Turkish study noted that medical error rates due to stress-related presenteeism among nurses increased by 2.3-fold compared with normal conditions [4]. Thus, developing targeted interventions to enhance ICU nurses’ psychological resilience and reduce presenteeism rates has become a critical pathway to improving nursing quality and patient safety [1–3].

Current intervention strategies for nurse presenteeism primarily focus on organizational support (e.g., flexible scheduling, psychological counseling) and individual behavioral training (e.g., stress management programs). However, randomized controlled trials (RCTs) indicate limited long-term efficacy of traditional interventions for ICU nurses: On one hand, the closed work environment and shift schedules of ICUs hinder the implementation of systemic organizational reforms; on the other hand, fragmented psychological training lacks neuroscientific theoretical foundations and fails to effectively regulate the physiological mechanisms of stress responses [5,10,11]. A recent qualitative study of Iranian emergency medical service providers revealed that healthcare workers rely on informal, pre-mission mental preparation, scene-specific risk management, adaptive coping (exercise, spirituality) and supportive peer communication to mitigate work stress, yet these strategies remain piecemeal and unsystematic [12]. Thus, evidence-based, structured and sustainable interventions—such as Mindfulness-Based Stress Reduction—are urgently needed to complement or replace existing fragmented approaches and effectively reduce ICU nurse presenteeism.

In recent years, mindfulness theory-based interventions have demonstrated unique advantages in occupational health due to their dual-pathway mechanisms of decentering and acceptance and commitment [10,13,14]. Mindfulness-Based Stress Reduction (MBSR), developed by Kabat-Zinn in 1979, aims to enhance awareness of present-moment experiences through structured practices (e.g., body scanning, mindful breathing, and nonjudgmental awareness training), thereby breaking the stress-emotion-cognition negative cycle [5,10,15]. Neuroimaging studies confirm that MBSR reduces amygdala activation and strengthens prefrontal-limbic system connectivity, improving stress response patterns [15,16]. In nursing, MBSR has been applied to reduce post-traumatic stress symptoms in emergency nurses and enhance work engagement among pediatric nurses [17,18]. For instance, a randomized controlled trial involving 80 nurses at Mashhad Psychiatric Hospital in Iran showed that an 8-week MBSR intervention reduced Perceived Stress Scale (PSS-10) scores by 37% and improved Compassion Fatigue Scale scores by 29% [17]. Additionally, a study of 119 intern nurses at a tertiary hospital in Beijing, China, found that online MBSR courses significantly increased psychological resilience (RSCA scores ↑21%) and mindfulness levels (FFMQ-15 total scores ↑18%)1515. These findings suggest that MBSR may alleviate occupational stress and reduce presenteeism by enhancing mindfulness traits, as assessed via the Five Facet Mindfulness Questionnaire (FFMQ), which includes observing, describing, acting with awareness, nonjudging, and nonreactivity [10,15,19].

Despite the demonstrated potential of MBSR in improving nurses’ mental health, existing research has two major limitations: First, most trials focus on general ward or student nurse populations, lacking cluster randomized controlled designs (cluster RCTs) tailored to ICU nurses, whose unique workloads and emotional exposures may influence intervention outcomes [11,20]; Second, outcome measures predominantly target psychological symptoms like anxiety and depression, neglecting direct assessment of presenteeism (quantified via SPS-6) [1,6,8]. Based on these limitations, this study proposes the following hypothesis: An MBSR intervention targeting ICU nurses will reduce presenteeism rates (SPS-6 scores) by improving Five Facet Mindfulness levels (FFMQ total and subscale scores), with clinically significant and sustainable effects under a cluster RCT design.

## 2 Method

### 2.1 Study design

The study utilized a cluster randomized controlled trial design. To ensure the independence of the intervention and control groups and reduce confounding factors, structured spatial separation was implemented via ward physical isolation [21].The clusters in this study were the floors on which the nurses worked (8th and 9th floors). An independent statistician, not involved in the recruitment or intervention process, prepared the list of eligible clusters and generated the randomization sequence using a computer – generated random number sequence to ensure unbiased allocation. To maintain allocation concealment, the randomization was performed using sealed opaque envelopes that were opened only after the participants had provided informed consent and completed the baseline assessments. The intervention group comprised the 8th floor cluster, while the control group included the 9th floor cluster. The two groups were well – balanced in terms of sex, age, education, and job title. The intervention group received an 8 – week Mindfulness-Based Stress Reduction (MBSR) program, whereas the control group received standard psychological counseling.

### 2.2 Participants

The trial was conducted from September 2021 to March 2022 in the general ICU of a tertiary general hospital in Sichuan Province, China. The hospital, with over 1,800 beds and more than 1,500 nurses, comprises an ICU with over 100 beds and a staff of more than 200 nurses. This hospital is representative of tertiary hospitals in China’s western region, where nurses work in a high-pressure environment due to heavy patient load and complex conditions.The participants were nurses identified in a prior cross-sectional survey as having high presenteeism (SPS-6 ≥ 16). Before the study, we thoroughly informed all potential participants of the study’s purpose, procedures, potential benefits and risks, and their right to withdraw at any time without penalty. Additional explanations were provided to those who needed them to ensure full comprehension. Written informed consent was obtained from all participants. The inclusion and exclusion criteria are shown in Table 1.

**Table 1:** Inclusion and exclusion criteria.

Inclusion Criteria	Exclusion Criteria
Nurses who have worked in ICU for more than 2 years and are able to work independently	Nurses with foreseeable personnel movements, pregnancy or impending retirement
Nurses with a licence to practise who are on duty and in employment	Nurses who are participating in other psychological interventions or have previously participated in Mindfulness-Based Stress Reduction training
Nurses who are able to understand the research process, give informed consent and participate voluntarily	Nurses with a previous diagnosis of mental illness.
Nurses with high presenteeism (according to SPS-6) in a previous cross-sectional survey.	

### 2.3 Introduction to the intervention methodology

Two trained psychological counselors implemented all study interventions. Regular supervision and feedback sessions were held with the counselors to ensure protocol adherence. Researchers also monitored participants’ task completion to guarantee protocol compliance.

The control group received routine psychological counseling, which centered on emotion regulation, psychological adaptation, and sleep management, and incorporated strategies from evidence-based practices such as cognitive-behavioral therapy (CBT). The counseling was conducted in a group format, once a week for 2.5 hours over eight weeks, aiming to support ICU nurses in managing work-related stress.

In the experimental group, a mindfulness therapist implemented an MBSR intervention. The intervention consisted of 8 weeks of intensive lectures and after-class practice, with intensive lectures once a week for 2.5 hours, including one hour of theoretical explanation, one hour of free practice under the guidance of the therapist, and 0.5 hours of sharing and discussion of feelings. After class, a WeChat platform was used to conduct follow-up assessments and provide advice, and a MBSR therapy practice logbook and practice audio were distributed at the end of each group lecture. The specific intervention content is provided in Table 2.

**Table 2:** Mindfulness-based stress reduction interventions.

Date	Themes	Contents and procedures	Homeworks
First week	Starting the Journey to Mindfulness	To introduce the origin and development of Mindfulness-Based Stress Reduction, the current situation of presenteeism among ICU nurses, the risk factors and the adverse effects of presenteeism. Lead nurses in practicing breath awareness and raisin exercises to enhance their focus and sensory awareness. Distribute the MBSR record book and the audio of the Positive Eating Exercise and teach the nurses how to fill it out and record it.	Follow the audio for Mindfulness Eating exercises, 6 times a week. Read the non-evaluation of the Seven Principles of Mindfulness.
Second week	The Power of the Moment	Explain the presence model. lead nurses in practicing body scanning. Invite nurses to share a pleasurable event.	Follow the audio for body scanning exercises, 6 times a week Read the Seven Principles of Mindfulness – Acceptance. Awareness of one pleasurable event per day and recording of it, 6 times per week.
Third week	Perceiving Inert-ial Reactions U-nder Stress	explain the inertial response under pressure. Invite nurses to share an unpleasant event. Lead nurses in practicing Mindfulness of Sound.	Follow the audio for Mindfulness Observation Sound Practice, 6 times a week. Read the Seven Principles of Mindfulness – Trust. Awareness of one unpleasant event per day and recording of it, 6 times per week.
Fourth week	Living with Str-ess-Aware StressResponse	Explain the pressure to respond with awareness. Lead nurses in practicing Mindfulness Standing Yoga and Mindfulness Walking.	Follow the audio for Mindfulness Standing Yoga practice, 6 times a week. Read the Seven Principles of Mindfulness -Patience.
Fifth week	Reclaiming the mind-body con-nection	explains reclaiming the mind-body connection and being present with discomfort. Lead nurses in practicing Mindfulness Lying Yoga	Follow the audio for Mindfulness Lying Yoga practice, 6 times a week. Read the Seven Principles of Mindfulness -The Pursuit of Non-Exertion.
Sixth week	Mindfulness and Compassion	Explain the importance of linking. Lead nurses in practicing Mindfulness Blessings	Follow the audio for Mindfulness Blessings practice, 6 times a week. Read the Seven Principles of Mindfulness – Letting Go.
Seventh week	Mindfulness and Communication	Explain the important role of communication. Lead nurses in practicing Mindfulness communication. Invite nurses to share a difficult communication.	Follow the audio for Mindfulness listening practice, 6 times a week. Read the Seven Principles of Mindfulness – Original intention. Awareness of one unpleasant event per day and recording of it, 6 times per week.
Eighth week	Mindfulness and Life	Review the seven principles of Mindfulness to reinforce the core concepts and practices learned over the eight weeks. Nurses are invited to share their eight weeks of learning on positive thinking. the end of the 8 weeks positive stress reduction program is not the end, but the beginning of the journey of Mindfulness in life, so that Mindfulness can be truly integrated into our lives and change our lives.	

### 2.4 Measurement tools

In this study, questionnaires were administered to the study participants before the intervention (T1), at the end of the intervention (T2), and 12 weeks after the end of the intervention (T3).

Presenteeism Scale: The Stanford Presenteeism Scale (SPS-6) was developed by Turpin et al. [22] was used. The scale consists of 6 items that mainly evaluate two aspects of the individual work process and work results. The items are scored on a 5-point scale, and items 5 and 6 are reverse scored. The total scores range from 6–30, and a higher score indicates greater impairment of productivity due to health problems. The Cronbach’s *α* coefficient of the scale was 0.86.

Mindfulness Scale: The Five Facet Mindfulness Questionnaire (FFMQ) was developed by Ruth et al. to measure the level of mindfulness [23]. The FFMQ consists of 39 items across 5 dimensions. The items are scored on a 5-point scale, and items 3, 5, 8, 10, 12, 13, 14, 16, 17, 18, 22, 23, 25, 28, 30, 34, 35, 38, and 39 are reverse scored. The total scores range from 39 ~ 195, with higher scores indicating a greater level of mindfulness among ICU nurses. The Cronbach’s *α* coefficient of the scale was 0.81, and the Cronbach’s *α* of each dimension was 0.70 ~ 0.88.

### 2.5 Procedure

#### 2.5.1 Blinding.

This study was not blinded because of the nature of the psychological intervention, which requires direct participation and trust, making blinding difficult. Nevertheless, data analysis was performed blinded to participant group assignments, thereby reducing potential data processing bias. Additionally, participants were cluster-randomized by floor (8th and 9th floors) to minimize contamination between the intervention and control groups.

#### 2.5.2 Contamination bias.

We attempted to minimize contamination bias in two ways. First, the nurses were grouped according to their floor to reduce the likelihood of participants in both groups sharing experiences. Second, before the intervention, all participants were told to avoid sharing project-related content with nurses outside their assigned groups.

#### 2.5.3 Sample size calculation.

The sample size calculation formula for this repeated measurement data is as follows:


$$\frac{{\left\{Z_{\left(\text{1}-\frac{\alpha }{\text{2}}\right)}+Z_{\left(\text{1}-\gamma \right)}\right\}}^{\text{2}}(\text{1}-\rho )\text{Var}\left({\text{Y}}_{ij}\right)}{\pi \left(\text{1}-\pi \right)\delta^{\text{2}}\sum_{j=\text{1}}^{n}{\left(\text{tj}-\overset{―}{\text{t}}\right)}^{\text{2}}}$$

This study compared the therapeutic effects of two methods by recording SPS-6 scores.The sample size was calculated using PASS 2021 software (Tests for Two Means in a Repeated Measures Design module). Pilot data showed SPS-6 scores of 12.75 ± 2.89 (*mean* ± *SD*) in the intervention group and 14.05 ± 2.65 (*mean* ± *SD*) in the control group, giving a mean difference (*D1*) of 1.3, a pooled standard deviation (*σ*) of 2.7, and a Cohen’s *d* effect size of approximately 0.47 (95% *CI* 0.05 ~ 0.89). The design included three repeated measurements per participant, a compound symmetry covariance structure, and a within-subject correlation (*ρ*) of 0.20. With a two-sided *α* of 0.05 and 80% power, the analysis indicated a requirement of 64 participants. Considering the 20% sample size dropout, the final required sample size is calculated to be at least 80 (40 per group).

#### 2.5.4 Data collection.

After obtaining ethical approval and written consent from the participants, two trained team members distributed electronic questionnaires to the ICU nurses in both groups before the intervention (T1), at the end of the intervention (T2), and 12 weeks after the intervention (T3). All survey contents were created and managed using Questionnaire Star, an online survey and questionnaire software, with each option set as a mandatory item. If the subjects had any questions during the study, on-site explanations were provided promptly to ensure the quality of each questionnaire.

#### 2.5.5 Statistical methods.

Data in this study were anonymized by removing all personally identifiable information and assigning each participant a unique code for data tracking and labeling. The link between codes and participants’ identities was stored separately and securely. Anonymized data were stored on password-protected computers accessible only to authorized personnel. These steps effectively protected participant privacy and ensured data integrity. Excel 2019 was used to create an electronic repository of the data. The data were verified and saved by two people. All statistical analyses were conducted using IBM SPSS (Statistical Package for the Social Sciences) Statistics, version 24.0.

In this study, we excluded participants who did not complete the entire intervention process. Data analysis was conducted using Per-protocol (PP) analysis, and no imputation or handling of missing data was performed.

For quantitative data, since the data meets the requirements of normality and homogeneity of variance distribution, *mean±standard* deviation is used for descriptive statistics, *t-test* and analysis of variance (*Welch test*) are used for difference testing; For count data, descriptive statistics use the number of cases and percentages for descriptive statistics, and *chi square test* or *Fisher’s* exact probability method for difference testing.

For the same indicator measured at different time points, the assumptions of normality of residuals and homoscedasticity were assessed and confirmed. *A linear mixed mode*l was used to analyze the interactions between different groups, time points, and their combination. Furthermore, *Bonferroni correction* was applied for post-hoc comparisons following the linear mixed model analysis to adjust for multiple comparisons and control the family-wise error rate. The two-tailed tests were used, with a *P-value* of less than 0.05 considered statistically significant.

#### 2.5.6 Ethical approval and registration.

The study was approved by the Ethical Review Committee at Deyang People’s Hospital (Ethics No. 2021-04-056-K01). This study followed the “Consolidated Standards of Reporting Trials (CONSORT)” guideline and was registered with the Chinese Clinical Trial Registry (Registration number: ChiCTR2400082570). For more information, see S1 File CONSORT Checklist and S1 Text Trial Protocol.

## 3 Results

### 3.1 Participant characteristics

**Fig 1:**
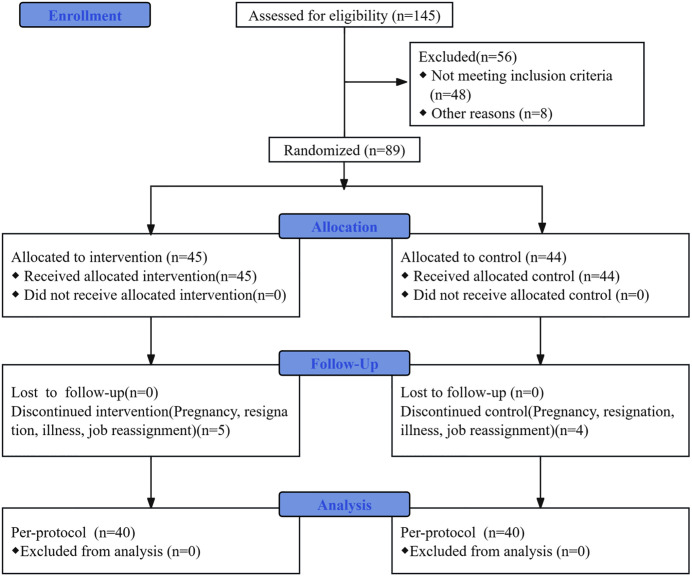
Consolidated standards of reporting trials (CONSORT) flow diagram.

The recruitment period for this study was from September 10, 2021, to September 15, 2021. Out of 145 nurses who participated in an earlier phase 1 study (cross-sectional survey) of ward nurses, 97 suffered from high presenteeism. However, eight of these nurses were excluded from the study due to previous involvement in positive stress reduction training, leaving 89 nurses who were invited to participate in the RCT. They were randomized into two groups – an intervention group and a control group (n = 45, 44). During the trial, 9 nurses withdrew: 2 due to pregnancy, 3 due to resignation, 2 due to illness, and 2 due to job reassignment. Thus, there were 40 nurses in each group (intervention and control) included in the final analysis. All participants in both groups completed all the interventions and assessments as per the protocol. The participant flow is shown in Fig 1.

Table 3 shows the characteristics of the participants. The two groups had no significant differences in baseline scores for any of the participants’ characteristics according to the chi-square test.Although there were differences in marital and education levels between the two groups, the chi-square test showed these differences were not statistically significant, indicating minimal impact on the study results. Moreover, the MBSR intervention, which enhances nurses’ mindfulness and stress reduction, is effective regardless of marital or educational background, further suggesting these factors did not substantially influence the outcomes.

**Table 3:** Comparison of general information of ICU nurses in two groups.

Participants’ Characteristics	Intervention Group		Control Group		*χ2 (df)*	*P-value*
	*n* = 40	(%)	*n* = 40	(%)		
**Gender**					0.000 (1)	1.000
Male	2	5.00	2	5.00		
Female	38	95.00	38	95.00		
**Age (Years)**					0.499 (3)	0.919
≤ 29	15	37.50	13	32.50		
30 −39	12	30.00	11	27.50		
40 −49	8	20.00	10	25.00		
≥ 50	5	12.50	6	15.00		
**Marital status**					4.599 (2)	0.100
Unmarried	12	27.50	10	25.00		
Married without children	3	7.50	10	25.00		
Married and having children	26	65.00	20	50.00		
**Education level**					2.400 (1)	0.121
College and below	7	17.50	13	32.50		
Undergraduate	33	82.50	27	67.50		
**Title**					0.298 (3)	0.960
Nurse	5	12.50	4	10.00		
Primary-Nurse	21	52.50	22	55.00		
Nurse-in-charge	10	25.00	9	22.50		
Associate Chief superintendent Nurse	4	10.00	5	12.50		
**Years of work experience in ICU (Years)**					0.311 (2)	0.856
2-5	11	27.50	9	22.50		
6-10	17	42.50	19	47.50		
≥ 10	12	30.00	12	30.00		
**Health condition**					0.313 (2)	0.855
Good	24	60.00	25	62.50		
Fair	12	30.00	10	25.00		
Poor	4	10.00	5	12.50		
**Chronic diseases**					0.105 (1)	0.745
Yes	6	15.00	5	12.50		
No	34	85.00	35	87.50		
**Night Shifts**					0.346 (1)	0.556
Yes	32	80.00	34	85.00		
No	8	20.00	6	15.00		

* indicates **P* *< 0.05. *χ² values* are presented with *degrees of freedom (df)* in parentheses.

### 3.2 Effect of mindfulness-based stress reduction training on SPS-6

**Fig 2:**
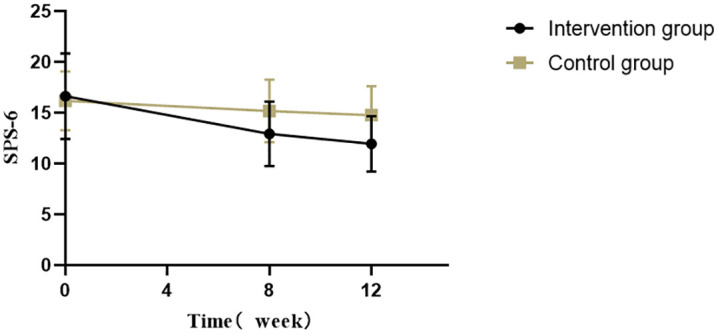
SPS-6 scores of two groups at three time points.

*A linear mixed model* was used to analyze the effects of combination and time on SPS-6, and the results showed that there were statistically significant differences in the interaction between different groups, time points, and groups and time; Further pairwise comparisons using Bonferroni correction showed that the SPS-6 score before intervention was significantly higher in the experimental group than at 8 weeks and 12 weeks after intervention, but there was no statistically significant difference in SPS-6 score between 8 weeks and 12 weeks after intervention; Within the control group, there was no statistically significant difference in SPS-6 scores before intervention, 8 weeks after intervention, and 12 weeks after intervention. Before intervention, there was no statistically significant difference between the experimental group and the control group. After 8 weeks of intervention, the SP6-S score of the experimental group was significantly lower than that of the control group, and the difference was statistically significant. After 12 weeks of intervention, the SPS-6 score of the experimental group was significantly lower than that of the control group, and the difference was statistically significant(see Table 4 & Fig 2).

**Table 4:** Comparison of SPS-6 scores at each time point before and after the intervention between the two groups of nurses.

Variable	Groups		*F*_main effect_	*F*_time_	*F*_Interaction_
	Intervention group (*n* = 40)	Control group (*n* = 40)			
**Time**			13.81	18.33	5.35
***P*-value**			<0.001	<0.001	0.006
**T1**	16.63 ± 4.22a*	16.18 ± 2.90a*			
**T2**	12.93 ± 3.17b*	15.18 ± 3.08a#			
**T3**	11.95 ± 2.73b*	14.75 ± 2.86a#			

Different letters (e.g., a, b, c) show pairwise comparison results within the same group at different time points. Same letters mean no significant difference; different letters mean a significant difference. Different symbols (e.g., *, #) show pairwise comparison results between groups at the same time point. Same symbols mean no significant difference; different symbols mean a significant difference. Bonferroni correction was applied to adjust p-values for multiple comparisons.

### 3.3 Effect of mindfulness-based stress reduction training on FFMQ

**Fig 3:**
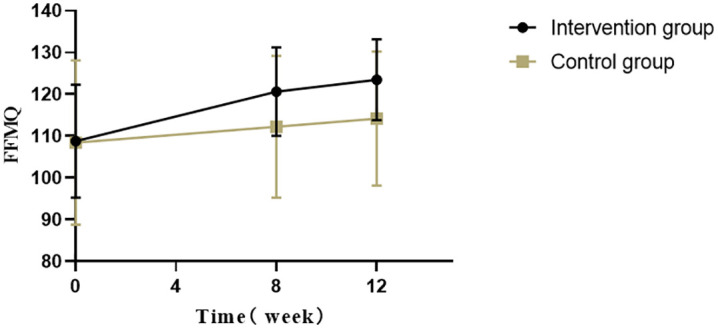
FFMQ scores of two groups at three time points.

*A linear mixed model* was used to analyze the effects of combination and time on FFMQ. The results showed that there were statistically significant differences among different groups and at different time points, but there was no statistically significant difference in the interaction between groups and time; Further pairwise comparisons using Bonferroni correction showed that in the experimental group, the FFMQ scores before intervention were significantly lower than those at 8 weeks and 12 weeks after intervention, but there was no statistically significant difference in FFMQ scores at 8 weeks and 12 weeks after intervention; Within the control group, there was no statistically significant difference in FFMQ scores before intervention, 8 weeks after intervention, and 12 weeks after intervention. Before intervention, there was no statistically significant difference between the experimental group and the control group; After 8 weeks of intervention, the FFMQ score of the experimental group was significantly higher than that of the control group, and the difference was statistically significant. After 12 weeks of intervention, the FFMQ score of the experimental group was significantly higher than that of the control group, and the difference was statistically significant(see Table 5 & Fig 3).

**Table 5:** Comparison of FFMQ scores at each time point before and after the intervention between the two groups of nurses.

FFMQ	Groups		*F*_main effect_	*F*_time_	*F*_Interaction_
	Intervention group (*n* = 40)	Control group (*n* = 40)			
**Time**			9.88	9.36	1.95
***P*-value**			0.002	0.001	0.150
**T1**	108.75 ± 13.55a*	108.40 ± 19.67a*			
**T2**	120.63 ± 10.63b*	112.20 ± 16.95a#			
**T3**	123.45 ± 9.75b*	114.15 ± 16.05a#			

Different letters (e.g., a, b, c) show pairwise comparison results within the same group at different time points. Same letters mean no significant difference; different letters mean a significant difference. Different symbols (e.g., *, #) show pairwise comparison results between groups at the same time point. Same symbols mean no significant difference; different symbols mean a significant difference. Bonferroni correction was applied to adjust p-values for multiple comparisons.

## 4 Discussion

This cluster randomized controlled trial investigated the effects of Mindfulness-Based Stress Reduction (MBSR) on presenteeism among ICU nurses, employing linear mixed models for multidimensional data analysis. The results demonstrated that MBSR significantly improved both presenteeism (measured by the Stanford Presenteeism Scale-6, SPS-6) and mindfulness levels (assessed via the Five Facet Mindfulness Questionnaire, FFMQ), with sustained effects observed over time. These findings provide empirical support for mental health interventions targeting ICU nurses while advancing the theoretical understanding of MBSR mechanisms. However, we found that the group × time interaction for total FFMQ scores was non-significant. This may be mainly due to the following factors: First, the routine psychological counseling received by the control group covered emotion regulation, psychological adaptation, and sleep management, and incorporated strategies from evidence-based practices such as cognitive-behavioral therapy (CBT), which may have indirectly enhanced specific mindfulness subdomains and reduced inter-group differences. Second, improvements in certain subscales may have been masked by the total FFMQ score. Third, there may be limitations in measurement sensitivity and statistical power. Future research could increase the sample size, use more sensitive measurement tools, and conduct a more in-depth analysis of the differences in the effects of the subscales.

Rooted in the integration of Eastern contemplative practices and modern psychology, MBSR operates through multifaceted mechanisms. Cognitively, it enhances metacognitive awareness, enabling nurses to identify and modify automatic negative thought patterns. ICU nurses chronically exposed to high-intensity stressors frequently enter a “burnout-forced attendance” cycle [10,24]. The improvements in FFMQ total scores, especially in the non-judging and acting with awareness subdomains, show that MBSR encourages open acceptance of workplace stressors. It reduces cognitive avoidance of negative experiences and lessens emotionally driven presenteeism [25,26]. Neuroimaging evidence corroborates this mechanism: MBSR strengthens prefrontal cortex regulation over amygdala hyperactivity, suppressing stress-reactive neural circuits [25], which aligns with the observed SPS-6 reduction trends. Physiologically, MBSR ameliorates chronic stress accumulation by balancing autonomic nervous system function. ICU nurses often exhibit sympathetic hyperactivation due to shift work and emergent responsibilities [27]. After the intervention, vagal tone likely improved, restoring sympathovagal balance [27,28]. This, in turn, eases physical symptoms like headaches and fatigue, which are key contributors to presenteeism [5,29]. Furthermore, mindfulness-induced relaxation responses reduce cortisol levels, disrupting the stress-inflammatory axis [13,30], providing a biological basis for improved occupational resilience.

While consistent with prior mindfulness interventions for healthcare workers, this study uniquely focuses on presenteeism in ICU nurses. Existing literature predominantly addresses burnout or anxiety/depression [5,10,24], whereas our use of SPS-6 as a primary endpoint highlights the modifiability of this underrecognized occupational health issue. Compared to general ward nurses, ICU nurses face heightened ethical decision-making pressures and greater emotional labor demands [24], rendering their presenteeism more vulnerable to emotional exhaustion. In this study, MBSR greatly improved the “describing difficulties” FFMQ subscale. This implies the intervention might boost emotional expression, curbing work-efficiency drops caused by emotional suppression [18,26]. This deepens our insight into healthcare workers’ emotional labor management. The sustained effects seen at the 12-week follow-up match MBSR’s neuroplasticity mechanisms. Long-term practice induces structural brain changes like increased insular gray matter density or default mode network reorganization [25], creating a “neuroprotective buffer” against stress [18]. Compared to single-session psychoeducation or short-term relaxation training, MBSR offers lasting protection via neuroplasticity, rather than merely modulating short-term stress responses [31]. This makes it particularly suitable for continuous high-pressure environments like ICUs [20,24]. Notably, in this study, the “acting with awareness” (FFMQ subscale) showed a higher late-stage increase than other dimensions. This aligns with the “mindfulness skill transfer” theory expectation. As practice deepens, nurses can naturally integrate the awareness developed in training into their daily workflow [18,32].

Clinically, this study informs evidence-based mental health strategies for healthcare institutions. MBSR has a dual benefit in reducing presenteeism. It lowers the risk of medical errors caused by presenteeism [5]and enhances nurses’ job efficacy by boosting their mindfulness levels [18,26]. Unlike traditional organizational support measures, such as shortening working hours, MBSR develops endogenous psychological resources that are more sustainable [11,28]. It is particularly worth highlighting that this study utilized a standardized 8-week program alongside daily practice, a design that has confirmed the viability of structured interventions within healthcare systems [10,20]. This approach provides an effective strategy for addressing the issue of fragmented training time that ICU nurses often encounter. The differential changes across FFMQ subscales imply that nurses with distinct traits may benefit from MBSR via a diverse pathway. For instance, individuals with a significant increase in the “observe” dimension may more easily alleviate physical symptoms through sensory awareness, while those with notable improvement in the “non-react” dimension tend to develop cognitive dissociation abilities [25,26]. This multidimensional mechanism supports the use of MBSR as a “core intervention module” for promoting the mental health of ICU nurses. Subsequently, it can be integrated with cognitive behavioral therapy or organizational reforms to form a multilevel intervention system [11,13,33].

In conclusion, this study systematically validates MBSR’s capacity to improve presenteeism and mindfulness in ICU nurses through cognitive restructuring, physiological regulation, and neuroplastic adaptation. These findings establish a scientific foundation for implementing evidence-based psychological interventions in high-stress clinical environments, ultimately enhancing caregiver resilience and patient safety.

## 5 Limitations

This study has several limitations. First, the study sample was restricted to ICU nurses in a tertiary hospital in Sichuan Province due to time and resource constraints. This may lead to selection bias and limit the generalizability of the findings. Future research could expand to multi-center ICU nurses to enhance feasibility and applicability. Second, the study primarily used subjective measures, which may affect the comprehensiveness of the results. Subsequent studies should assess the effectiveness of MBSR across multiple dimensions, including objective physiological, behavioral, and imaging indicators. Lastly, the relatively short follow-up period of 12 weeks might be insufficient for evaluating the long-term sustainability of the intervention effects. Long-term follow-up studies are necessary to examine the durability of MBSR’s impact on presenteeism and mindfulness in ICU nurses.

## 6 Conclusion

This study robustly demonstrates that Mindfulness-Based Stress Reduction (MBSR) can effectively enhance nurses’ mindfulness levels and reduce presenteeism, offering hospital administrators a practical intervention tool. Specifically, administrators can incorporate MBSR into hospital training programs by conducting 2.5-hour-long weekly training sessions for 8 weeks to help nurses develop awareness and acceptance of their present-moment experiences. This enables them to better focus on their inner feelings and physical state, thus efficiently handling work stress. Consequently, this approach not only improves nurses’ work efficiency and mental health but also elevates patient care quality, fostering a safer and more efficient healthcare environment.

## Supporting information

S1 File CONSORT checklist. (PDF)

S1 Text Trial protocol. (PDF)
