# Supplementary material for: The effect of mindfulness-based stress reduction on presenteeism among ICU nurses: A cluster randomized controlled trial
Source: PLoS One. 2025 Oct 28;20(10):e0334825. doi: 10.1371/journal.pone.0334825 (PMC12561986; doi:10.1371/journal.pone.0334825)
Supplement: S1 Text — (PDF) [file pone.0334825.s002.pdf]

A clinical research protocol for the effect of Mindfulness-Based Stress Reduction on presenteeism among ICU nurses

(Interventional clinical research)

Research unit: Deyang People's Hospital

Leader (signature): Xiaoli Liu

Department: Department of Critical Care Medicine

Tel: 13548260274

Team Leader Unit: None

Participating units: none

Years of study: August 2021 to March 2022

Version No.: V1.0

Version date: August 15, 2021

Formula Case Abstract Summary

**Study design (optional)**	☐**Case-control studies** ☐**Cross-sectional study**<br>☐ **Cohort studies** ☐**Other:**<br>☑**Randomized controlled studies**<br>☐**Application of blinding**
**Type of study (Please check according to project type)**	**(Category A: High risk)**<br>□ Gene editing research<br>□ Cell Therapy Research<br>□ Implantable medical device research (including 3D printing)<br>□ Class III new clinical technology (exact safety and efficacy, technical difficulty and high risk)<br>□ Special population studies (children, pregnant women, mentally retarded, mentally challenged subjects, etc.)<br>□ Superficial studies (□ Superficial indications □ Multi-route of administration □ overdose □ over-age □ Ultra contraindications<br>□ Super Population □ Other, please specify:)<br>□ Ultra Device Specification Study (□ Ultra Indications □ Scope of use<br>□Super contraindications □ Superpopulation □ Other, please specify:)<br>□ Other (As determined by the researcher, please specify:)<br>**(Category B: Medium risk)**<br>□ Post-market biologics studies (prophylactic and therapeutic)<br>□ Post-market therapeutic vaccine studies<br>□ Post-market rare disease drug research<br>□ Class II New Clinical Technology (exact safety and efficacy, certain technical difficulties, certain medical and ethical risks)<br>□ Other (As determined by the researcher, please specify:)<br>**(Category C: low risk)**<br>□ Research on marketed drugs (including non-registered categories such as chemicals and generics)

	□ Research on marketed devices (including AI, imaging software)<br>□ Type I new clinical technologies (medical technologies with precise safety and efficacy, low technical difficulty and little or no ethical isk)<br>☑ Other (researcher judgment, please note: Positive thought stress reduction is a more established method of stress reduction, almost (No ethical risk)
**Total number of cases**	80 cases
**Risk/benefit analysis**	Benefit: By participating in this research, participants can obtain multiple benefits: master Mindfulness-Based Stress Reduction (MBSR), learn to use Positive Thought Stress Reduction (PTSR) for self-psychological regulation, reduce the occurrence of hidden absenteeism, and further improve work efficiency..<br>Risks: During the research process, several risks may arise, including the aggravation of pre-existing health problems (or psychological problems), the inducement of psychological trauma, and the development of other new psychologically related problems, etc.
**risk judgment**	☑Not greater than minimal risk □ Greater than minimum risk<br>Minimal risk: the likelihood and magnitude of the expected risk in a trial is no greater than in daily life, or in the performance of a routine physical examination.
**Duration of the study**	August 1, 2021 to February 28 ,2022

I. Background of the study

Presenteeism is defined as the behavior of a person who is present at work but has a low workload and inefficiency. It has become a significant concern in various professions, particularly in healthcare settings. In the nursing profession, presenteeism is especially prevalent, with incidence rates 3 to 4 times higher than those in other occupations.This issue is further exacerbated among intensive care unit (ICU) nurses, who face unique challenges due to the high-stress, high-demand nature of their work environment. Studies have shown that the prevalence of presenteeism among ICU nurses ranges from 55.26% to 55.40%.

The consequences of presenteeism in the nursing workforce are multifaceted. Several studies have shown that presenteeism among ICU nurses is significantly associated with the number of patient falls, patient medication errors, and nursing quality and safety, thereby leading to increased health care costs.Given the substantial impact of presenteeism , it is crucial to identify effective interventions to mitigate this issue. However, current research on presenteeism among nurses has predominantly focused on phenomenological studies, with limited exploration of intervention strategies, which would primarily involve physical interventions. Traditional interventions, such as physical health programs or basic psychological counseling, have shown limited success in addressing the complex nature of presenteeism.

II. Purpose of the study

1. To evaluate the effect of an 8-week Mindfulness-Based Stress Reduction (MBSR) training on presenteeism among ICU nurses.

2. To provide implications for subsequent exploration and mitigation of nurses' presenteeism.

III. Research design, methodology

1. Research design

The study utilized a cluster randomized controlled trial design. A study compared the therapeutic effects of two methods, recording SPS-6 scores at T1, T2, and T3, with an estimated intervention group result of 12.75 ± 2.89 and a control group result of 14.05 ± 2.65. After literature review, it was assumed that the correlation of repeated measurements was 0.2. The inspection level is 0.05 on both sides, and the inspection efficiency is 80%. PASS software is used for sample size calculation, and the final required sample size is 64. Considering the 20% sample size dropout, the final required sample size is calculated to be at least 80.

2. Research methodology

A mindfulness-based intervention program grounded in the MBSR theoretical framework and standard curriculum was developed to address presenteeism among ICU nurses. The General

Information Scale, Stanford Presenteeism Scale (SPS-6), and Five Facet Mindfulness Questionnaire (FFMQ) were administered before and after the intervention to evaluate changes in presenteeism and mindfulness.

IV. Case selection

1. Inclusion Criteria

① Nurses who have worked in the ICU for 2 years or more and are able to work independently;

② Nurses with a licence to practise who are on duty and in employment;

③ Nurses who are able to understand the research process, give informed consent and participate voluntarily;

④Nurses with high presenteeism (according to SPS-6) in a previous cross-sectional survey.

2. Exclusion criteria

① Nurses with foreseeable personnel movements, pregnancy or impending retirement;

②Nurses who are participating in other psychological interventions or have previously participated in Mindfulness-Based Stress Reduction training;

③Nurses with a previous diagnosis of mental illness.

3. Criteria for termination of research

The executive operation of this research project will be strictly in accordance with the norms to complete the research on the application of Positive Mindfulness Stress Reduction in alleviating the hidden absenteeism of ICU nurses, but this process may cause some adverse reactions, such as: aggravation of the existing problems, inducing psychological trauma, and generating other psychologically related problems, and so on. If any of these problems occur, the research participants should tell the researcher immediately, and the researcher will deal with the discomfort that occurs, and the researcher has the right to withdraw from this study at any time during the research process.

V. Alternative diagnostic and therapeutic options

Professional counseling and psychotherapy

VI. Test items and test points

Before, after, and 12 weeks after the Mindfulness-Based Stress Reduction

VII. Efficacy assessment criteria

Using The Stanford Presenteeism Scale (SPS-6), The Five Facet Mindfulness Questionnaire (FFMQ) and statistically analyzed to determine if there was a difference.

VIII. Observation, recording and disposition of adverse events

Expected adverse events: aggravation of existing problems, induction of psychological trauma, generation of other psychological related problems, etc. When the above problems occur, the research subjects should tell the researcher immediately, and the researcher will immediately ask a professional psychotherapist to provide psychological counseling and treatment, and at the same time, make relevant records, including the treatment measures, the way and time of the follow-up visit.

IX. Quality control and quality assurance of research

To ensure research standardization, data reliability and goal achievement, a special research team is established, consisting of 2 psychotherapists, 2 core psychological care members and 2 postgraduates, with responsibilities as follows:

Questionnaire Management: Design and develop questionnaires, coordinate distribution and collection, screen invalid questionnaires, and ensure data quality.

Program Formulation: Based on research purposes and subject characteristics, develop operable mindfulness-based stress reduction programs, clarifying processes, duration, methods and evaluation criteria.

Community Operation: Establish a mindfulness-based stress reduction WeChat group, release guidance content, track participation via check-in supervision, and improve compliance.

Progress Control: Track the progress of each research link, promptly address issues (e.g., delayed questionnaire collection, subject dropout), and ensure progress as planned.

X. Data security monitoring

Clinical studies will have appropriate data safety monitoring programs based on the level of risk. All adverse events are recorded in detail, handled appropriately and followed up until properly resolved or stabilized. Serious adverse events and unintended events will be reported in a timely manner to the Ethics Review Committee, the competent authorities, the sponsor, and the Drug Administration in accordance with the regulations; the principal investigator will conduct a cumulative review of all adverse events on a regular basis, and convene a meeting of the investigators to assess the risks and benefits of the study when necessary; double-blind trials may be unblinded on an emergency basis when necessary in order to ensure the safety and rights of the subjects.

XI. Statistical methods

For quantitative data, since the data meets the requirements of normality and homogeneity of variance distribution, mean±standard deviation is used for descriptive statistics, t-test and analysis of variance (Welch test) are used for difference testing; For count data, descriptive statistics use the

number of cases and percentages for descriptive statistics, and chi square test or Fisher's exact probability method for difference testing.

For the same indicator measured at different time points, the assumptions of normality of residuals and homoscedasticity were assessed and confirmed. A linear mixed model was used to analyze the interactions between different groups, time points, and their combination. Furthermore, Bonferroni correction was applied for post-hoc comparisons following the linear mixed model analysis to adjust for multiple comparisons and control the family-wise error rate. The two-tailed tests were used, with a P-value of less than 0.05 considered statistically significant.

XII. Ethical Principles and Requirements for Clinical Research

Ethical Principles and Requirements for Clinical Research Clinical studies will follow the Declaration of Helsinki of the World Medical Assembly and the National Health and Family Planning Center of the People's Republic of China.The Ethical Review of Biomedical Research Involving Human Beings by the Ethics Review Committee and other relevant regulations specify the implementation of the principlesandrequirementsofinformed consent, protection of privacy, research free of charge and compensation, control of risks, protection of special subjects and compensation for research-related damages. Prior to the commencement of the study, the trial protocol was approved by the Ethical Review Committee before the clinical study was implemented. Prior to the enrollment of each subject in the study, it is the responsibility of the investigator to provide the subject or/and his/her legal representative with a complete and comprehensive description of the purpose, procedures, and possible risks of the study, and to sign a written informed consent form, and to let subjects know that their participation in the clinical research study is entirely voluntary, that they may refuse to participate or withdraw from the study at any time during any phase of the trial without discrimination or reprisal, and that their medical treatment and rights will not be affected. rights will not be affected. The informed consent form should be retained as a clinical research document for record keeping, and the privacy and confidentiality of the subjects' data should be effectively protected.
